# Mutations in the Gene *DNAJC5* Cause Autosomal Dominant Kufs Disease in a Proportion of Cases: Study of the Parry Family and 8 Other Families

**DOI:** 10.1371/journal.pone.0029729

**Published:** 2012-01-03

**Authors:** Milen Velinov, Natalia Dolzhanskaya, Michael Gonzalez, Eric Powell, Ioanna Konidari, William Hulme, John F. Staropoli, Winnie Xin, Guang Y. Wen, Rosemary Barone, Scott H. Coppel, Katherine Sims, W. Ted Brown, Stephan Züchner

**Affiliations:** 1 New York State Institute for Basic Research in Developmental Disabilities, Staten Island, New York, United States of America; 2 Department of Pediatrics, Albert Einstein College of Medicine, Bronx, New York, United States of America; 3 Hussman Institute for Human Genetics, University of Miami Miller School of Medicine, Miami, Florida, United States of America; 4 Department of Pathology, Massachusetts General Hospital, Boston, Massachusetts, United States of America; 5 Neurogenetics DNA Diagnostic Laboratory, Center for Human Genetics Research, Department of Neurology, Massachusetts General Hospital, Boston, Massachusetts, United States of America; 6 Harvard Medical School, Boston, Massachusetts, United States of America; Charité Universitätsmedizin Berlin, NeuroCure Clinical Research Center, Germany

## Abstract

**Background:**

The Neuronal Ceroid Lipofuscinoses (NCL) comprise at least nine progressive neurodegenerative genetic disorders. Kufs disease, an adult-onset form of NCL may be recessively or dominantly inherited. Our study aimed to identify genetic mutations associated with autosomal dominant Kufs disease (ADKD).

**Methodology and Principal Findings:**

We have studied the family first reported with this phenotype in the 1970s, the Parry family. The proband had progressive psychiatric manifestations, seizures and cognitive decline starting in her mid 20 s. Similarly affected relatives were observed in seven generations. Several of the affected individuals had post-mortem neuropathological brain study confirmatory for NCL disease. We conducted whole exome sequencing of three affected family members and identified a pLeu116del mutation in the gene *DNAJC5*, which segregated with the disease phenotype. An additional eight unrelated affected individuals with documented autosomal dominant or sporadic inheritance were studied. All had diagnostic confirmation with neuropathological studies of brain tissue. Among them we identified an additional individual with a p.Leu115Arg mutation in *DNAJC5*. In addition, a pAsn477Ser change in the neighboring gene *PRPF6*, a gene previously found to be associated with retinitis pigmentosa, segregated with the ADKD phenotype. Interestingly, two individuals of the Parry family did report visual impairment.

**Conclusions:**

Our study confirmed the recently reported association of *DNAJC5* mutations with ADKD in two out of nine well-defined families. Sequence changes in *PRPF6* have not been identified in other unrelated cases. The association of vision impairment with the expected PRPF6 dysfunction remains possible but would need further clinical studies in order to confirm the co-segregation of the visual impairment with this sequence change.

## Introduction

Neuronal Ceroid Lipofuscinoses (NCL), also referred to as Batten Disease, are a group of at least 9 genetic disorders, characterized by progressive neurodegeneration and early death. A unifying feature of these devastating conditions is the lysosomal accumulation of autofluorescent lipopigment in neuronal cells [1], [2]. Electron microscopy (EM) studies of peripheral cells demonstrate specific storage inclusions in the lysosomes in some of these patients. Adult-onset NCL, also referred to as Kufs disease is classified into two categories with autosomal recessive (MIM 204300) or dominant (MIM162350) inheritance. In the majority of families with recessively inherited Kufs disease, mutations in the gene *CLN6* that are usually associated with early-onset NCL were recently reported [3]. No genetic defect associated with Autosomal-Dominant Kufs disease (ADKD) was known until very recently. An article describing mutation in the gene *DNAJC5* in patients with ADKD by Noskova et al. [4] was published while the current study was at its final stage. Noskova and colleagues reported two mutations in *DNAJC5* in five unrelated families or single individuals out of total of 20 studied individuals/families. Seven of the mutation-negative individuals/families studied by this group did not have documented family history so it was not clear if recessive inheritance was ruled out for these families. In addition, five of the families were reported with recessive inheritance. Most importantly, for the families negative for the *DNAJC5* mutations no evidence of neuropathological confirmation of the diagnosis was presented. Kufs disease is a disorder presenting with rather non-specific features of progressive neurodegeneration including seizures, psychiatric manifestations and progressive dementia. It is often difficult to be certain in this diagnosis without observing, usually postmortem, characteristic changes in neuronal cells. Here we report our own approach that led to the identification of *DNAJC5* mutations in two families, thereby confirming the recent study by Noskova et al [4]. We also present detailed clinical up-to-date on the proband of the Parry family. Finally, we estimate the proportional contribution of *DNAJC5* changes to all families with ADKD.

## Methods

### Objectives

To identify genetic mutations segregating with the phenotype in the originally reported family with ADKD.

To study additional, unrelated individuals, and estimate the mutations prevalence in affected individuals.

### Subjects

#### A. The Parry family

The family studied here was first reported in the 1970s [5]. Since it is the first family reported with this disorder, the ADKD is often referred to as the Parry type or Parry disease.

**Figure 1:**
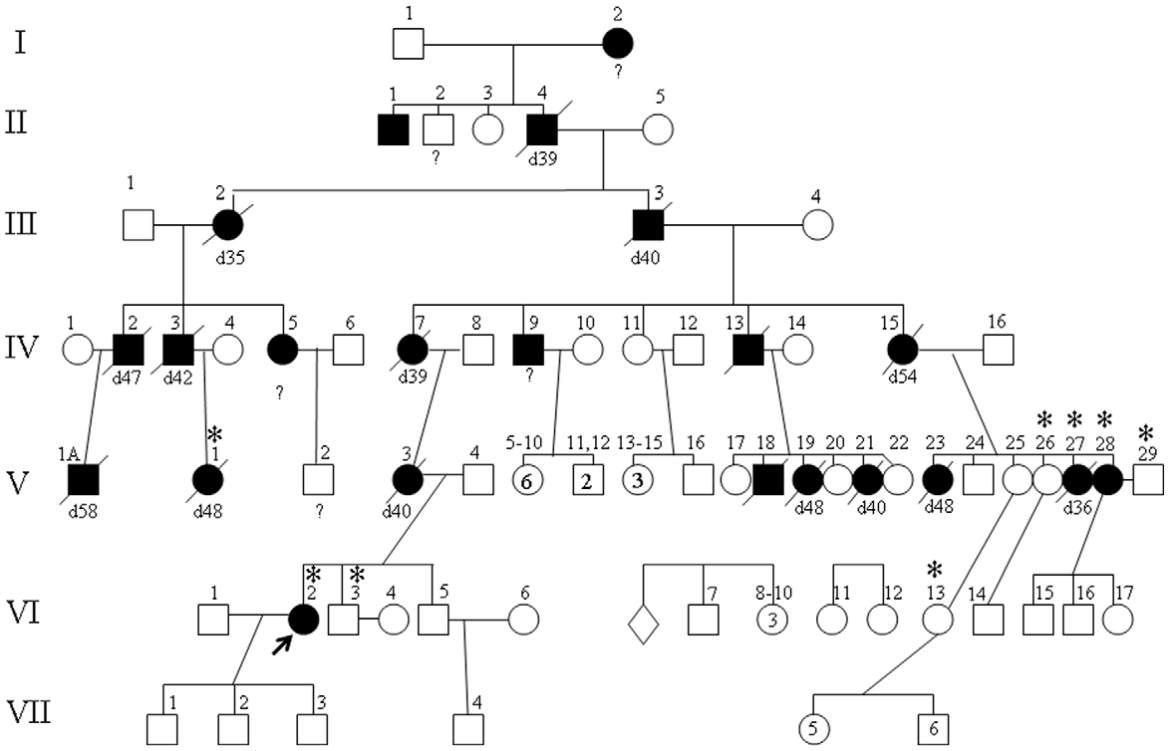
Updated pedigree of the Parry family. Individuals included in this study are indicated with star symbols on top of individual's number. The proband is marked with an arrow. One of the individuals in generation V was marked V-1A in order to match the individual numbers with the ones in the initially published Parry pedigree, reference 5.

Proband: The proband (individual VI-2 from the Parry pedigree in Figure 1) is a 49 year-old woman, who was in good health until her mid 20 s. At the age of 26 years she had several evaluations by different clinicians because of increasing irritability and obsessive-compulsive manifestations. She was worried that her house was not clean, was seen frequently washing her hands before touching her three children and quit her job because she believed that she might contract HIV from objects at work. Her EEG at that time was moderately abnormal with generalized recurrent brief bursts of moderate to high voltage 4–6 Hz slow waves, often with a semi-rhythmic pattern, and admixed sharp forms. On psychiatric assessment she appeared anxious and depressed, but she had intact memory. Otherwise her examination was normal. At the age of 32 years she had her first generalized seizure. She had normal brain CT scan at the age of 33 years and normal brain MRI at the age of 34 years. After her first seizure episode she gradually developed memory loss and an ataxic gait. At the age of 49 years, about every month around the time of her period, she had 1–2 day periods of multiple seizure events. She was able to ambulate with a cane. Her short-term memory was affected, but her long-term memory was relatively spared. Starting in her 40 s she complained of seeing “yellow blinding lights”. This complaint has progressively increased in frequency. Ophthalmologic examinations have been normal up until the final reported examination at the age of 34 years.

Other family relatives from the Parry family: All affected individuals from the family had a history of progressive seizure disorder, ataxic gait, and progressive dementia, with the first manifestations typically starting in their 20 s or 30 s. We have compiled information on 7 generations of the individuals of this family and matched the individuals' numbers with the ones reported in reference 5. Of interest is that one of the originally reported affected relatives (individual IV-2) had complaints similar to the proband of a “visual aura of bright spots” beginning at the age of 39 years.

The proband's mother died at the age of 40 years with clinical diagnosis of Kufs disease and a brain autopsy revealed neuronal autofluorescent inclusions thus supported the clinical diagnosis. Detailed information was previously reported in three individuals of this family: IV-2, IV-3 and IV-7 [5]. Brain autopsy results were previously reported in individuals IV-2 and IV-3, and both were confirmatory for NCL disease. The proband's clinically unaffected brother (individual VI-3) had a normal EEG examination and normal neurological examination at the age of 22 years.

Seven individuals from this family in addition to the proband were included in our study. All unaffected individuals studied were older than 32 years in order to avoid including preclinical individuals in our analysis.

#### B. Other individuals studied

**Figure 2:**
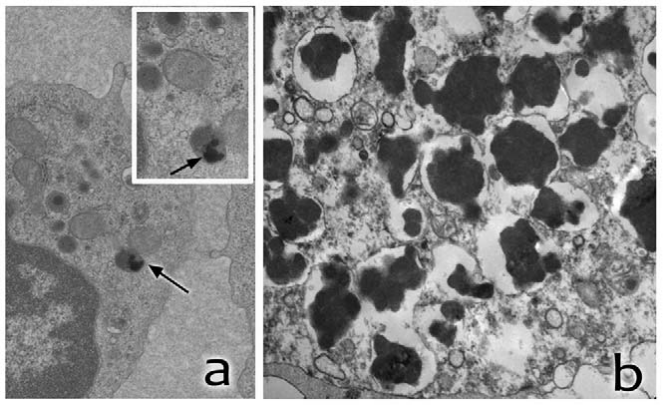
Laboratory studies of some of the individuals studied. a) EM study of peripheral lymphocytes of the proband of the Parry family showing storage inclusions with morphology of GROD (arrows). Higher magnification of the area is shown in the upper right corner. b) Neuropathology study of the father of individual KUF01CH showing neuronal accumulation, throughout the CNS, of PAS-positive material with strong, broad-range autofluorescence.

Eight additional individuals/families were included in the study. Four of them were with documented familial, autosomal-dominant inheritance and four were sporadic. All of them presented with a progressive neurodegenerative disorder with manifestations starting between the ages 16 and 35 years, and consisting of seizures, progressive dementia, unstable gait and behavioral changes, sometimes including frank psychiatric manifestations. No vision deficits were reported in these individuals. Confirmation of the diagnosis of Kufs disease was made in all individuals with neuropathological study of brain tissue in the proband or in affected family relative. Summary of the clinical manifestations of the studied individuals is shown on Table 1. Results of neuropathology and EM studies of peripheral cells in some of the individuals are shown on Figure 2. The results of laboratory studies are summarized on Table 2. Patient KUF04SR was previously reported [6]. This patient was also included in the study of Noskova et al [4].

**Table 1:** Clinical manifestations in probands from the families or sporadic affected individuals included in the study.

Proband ID	Clinical summary	Family history
P-VI-2 (Parry)	See text	See Figure 1and reference 5
BD 319	The early growth and development were normal. Seizures and gradual dementia started in mid 30 s. Interictal EEG was abnormal. No vision problems were reported. Neuropsychological testing revealed borderline cognitive functioning. Tendencies for denial and presenting himself in an overly favorable light were noted on psychological evaluation. Difficulties speaking, ataxia and involuntary movements developed in early 40 s. At 46 he had difficulties swallowing. Patient died at the age of 54 years.	No affected family relatives were reported
BD 303	At the age of 32 started having headaches, memory problems, difficulties speaking and coordination problems. No vision impairment was reported. Interictal EEG was abnormal, but no clinical seizure activity was observed. Brain MRI showed diffuse brain atrophy. Patient died at the age of 36.	This patient's father, paternal grandmother and brother were affected with similar progressive neurodegenerative disorder and died in their 30 s.or early 40 s
BD 160	In her mid 20 s this individual started having difficulties speaking, ataxia and seizures. The patient died at the age of 38.	The patient's brother and father had similar manifestations
KUF01CH	At 38 years of age this patient developed myoclonus and anxiety disorder. The interictal EEG was normal.	The patient's father developed involuntary movements, ataxia and memory loss in his 70 s
KUF04SR	Absence seizures started at the age of 5, episodic tremors started in the 20 s, generalized seizures since 28 and intellectual decline documented at 31. See also reference 6.	Autosomal-dominat (see reference 6)
BD-AW	This individual died at the age of 26 from progressive neurodegenerative disorder starting in her 20 s.	No other affected relatives were reported
BD-SS	Learning, speaking and walking difficulties and involuntary movements started at the age of 15. No vision impairment was reported. Seizures started at 23. Brain MRI showed cerebral atrophy.	No other affected relatives were reported
BD-185	Seizures and memory loss started at 16.	No other affected relatives were reported

**Table 2:** Laboratory, neuropathology studies and DNAJC5 mutation status of probands from the families included in the study.

Proband ID	Peripheral cell studies	Neuropathology studies	DNAJC5 gene change	PRPF6 gene change
P-VI-2 (Parry)	See text	See text and reference 5	pLeu116del	pAsn477Ser
BD-319	Not available	Brain biopsy at the age of 38: microscopy showed neuronal storage material that stained intensely with PAS stain and was autofluorescent under fluorescent light.EM* of brain tissue revealed GROD** in the neuronal cytoplasm	pLeu115Arg	negative
BD-303	EM in fibroblasts showed curvilinear bodies and lipofuscin granules	Post mortem brain neuropathology reportedly confirmed the diagnosis of Kufs disease	negative	negative
BD-160	EM demonstrated GROD inclusions in fibroblasts.	Brain autopsy showed GROD inclusions in neuronal cells.	negative	negative
KUF01CH	Rectilinear and GROD in peripheral lymphocytes (Figure 2)	Postmortem neuropathology study of the patient's father showed PAS positive inclusions and granular osmiophilic inclusions in neuronal cells in electron microscopy	negative	negative
KUF04SR	Not available	Brain neuropathology: microscopy: PAS positive, autofluorescent neuronal inclusions; electron microscopy: osmiophilic inclusions with rectilinear or curvilinear morphology	negative	negative
BD-AW	Not available	Postmortem electron microscopy study of brain tissue showed fingerprint and curvilinear inclusions	negative	negative
BD-SS	Normal study of peripheral lymphocytes and fibroblasts at the age of 24.	Brain biopsy: microscopy: PAS positive and autofluorescent storage material. EM: positive for osmiophilic neuronal inclusions.	negative	negative
BD-185	Not available	Brain neuropathology (brain biopsy) PAS positive and autofluorescent neuronal inclusions. EM showed osmiophillic inclusions	negative	negative

*EM = Electron microscopy;

**GROD = Granular osmophilic deposits.

The gene *CLN6* that was previously reported in association with autosomal-recessive Kufs disease was sequenced in all the sporadic individuals studied and no mutations in this gene were identified.

### Description of Procedures or Investigations taken

#### A. EM studies of peripheral cells

We have conducted EM studies of peripheral cells of the proband of the Parry family at three different ages, and of individual V-28 of the same family in order to monitor for NCL-specific lysosomal inclusions as previously described [2].

#### B. Exome sequencing

Enrichment of coding exons and flanking intronic regions was performed on three individuals of the pedigree in Figure 1: VI-2, V-1 and V-28. We applied the SureSelect Human All Exon 50 Mb kit (Agilent) following the manufacturer's standard protocol. The kit encompasses coding exons annotated by the GENCODE project, CCDS, and RefSeq databases and also incorporates a number of non-coding RNAs. The protocol allowed for integration of Illumina's Hiseq2000 adapter sequences and indexed multiplexing of multiple samples per flow cell lane. Enriched DNA was subjected to standard sample preparation for the Hiseq2000 instrument (Illumina).

#### C. Sanger Sequencing

Sanger sequencing was performed to confirm existence and segregation of identified variants and to screen for additional mutations in the two candidate genes *PRPF6* and *DNAJC5*. For these genes all exons and flanking intronic sequences were amplified on Applied Biosystems (ABI) Veriti 96-well Fast Thermal Cyclers using a touchdown protocol. Polymerase chain reaction (PCR) purification was completed with QuickStepTM2 SOPE resin (Edge BioSystems). Sequencing was performed using ABI BigDye Dye Terminator Cycle Sequencing Kit on an ABI 3730 sequencer. Sequence traces were analyzed using Sequencher (version 4.8; Gene Codes Corporation).

#### D. Bioinformatics Analysis

Raw sequencing data were analyzed using version 1.7 of the Illumina analysis pipeline. Sequence reads were aligned with the MAQ software. MAQ is a widely used short read mapping software for next-generation sequencing data. MAQ first aligns reads to reference sequences and then calls consensus genotypes based on a statistical model, which maximizes the posterior probability and calculates a quality score at each position along the consensus (http://maq.sourceforge.net/index.shtml).

Variants were called with MAQ applying the standard parameters. All variants were submitted to SeattleSeq for further categorization into coding, noncoding, and novel single-nucleotide polymorphisms (SNPs). SeattleSeq is a genomic annotation server that provides batch annotation of SNPs (single-nucleotide polymorphisms), both known and novel (http://snp.gs.washington.edu/SeattleSeqAnnotation). Resulting data were converged, filtered, and ranked by Genomic Evolutionary Rate Profiling (GERP) conservation scores.

#### E. Genotyping and haplotype analysis

Genotyping of the DNAJC5 locus was performed with Sanger sequencing. The genotypes were phased based on the segregation pattern within the pedigree and haplotypes were constructed from consecutive single nucleotide variants. Disease co-segregating haplotypes were compared to the published alleles in Noskova et al.

### Ethics

This study was conducted according to protocols approved by the Institutional Review boards of the New York State Institute for Basic Research in Developmental Disabilities and the Institutional Review Board of Massachusetts General Hospital. The study was conducted according to principles of the Declaration of Helsinki. All subject studied signed an informed consent for study participation.

## Results

### Electron microscopy studies

Lysosomal inclusions in peripheral cells are typically observed in certain types of NCL patients. The proband of the Parry family had three EM studies on peripheral lymphocytes and one study of fibroblast cells. The studies of peripheral lymphocytes were done at 26, 34 and 36 years of age. Two of the studies were negative for lysosomal inclusions while one study of peripheral blood cells at the age of 34 years revealed granular osmiphilic deposits (GROD) and curvilinear lysosomal inclusions in only two out of 112 cells studied. The clinically affected individual V-28 from the same family was also studied with electron microscopy at the age of 38 years. Her peripheral lymphocytes were negative for lysosomal inclusions.

### Exome sequencing and genetic studies

We performed exome sequencing on three affected family members of the Parry family. After alignment and variant calling 30,148 coding single nucleotide variants were identified across all individuals. Filtering for segregation of non-synonymous and heterozygous variants reduced this number to 14,683 and 911 changes respectively. Only 111 changes were not reported in dbSNP v132 and 8 changes had a GERP conservation score above zero. These changes represented top candidates for this very rare disease. Sanger sequencing found only one variant completely segregating in the pedigree. This change c.A1430G, represented a missense variant in the gene *PRPF6*, Asn477Ser. The variant was highly conserved (GERP score 5.11; PhastCons: 0.957) and the change was not present in 2,100 control individuals.

Indel analysis revealed 5,464 variants segregating between the three exomes. Of these 109 changes were coding or affected a splice site and only four indels were not in dbSNP v132. As confirmed by Sanger sequencing, a deletion in the gene *DNAJC5*, c.346_348delCTC, p.Leu116del, was the only indel completely co-segregating with the phenotype in the pedigree (Fig. 1).

**Figure 3:**
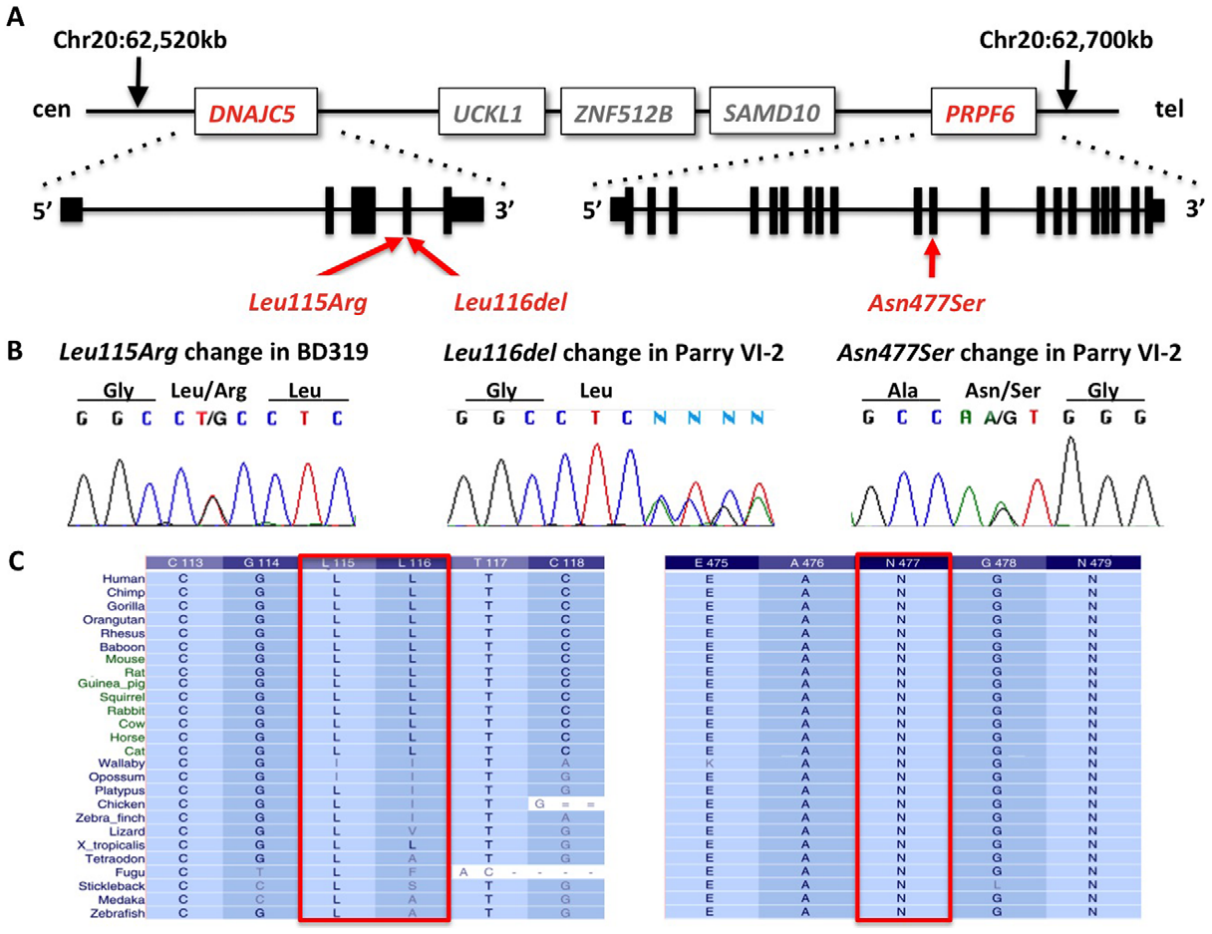
Identification of DNA variants in *DNAJC5* and *PRPF6*. a) Schematic of the region on chromosome 20 containing *DNAJC5* and *PRPF6* in close proximity. b) Sanger sequence traces of the identified segregating variants in *DNAJC5* and *PRPF6*. c) The two very rare changes identified in the Parry family Leu116del (in *DNAJC5*) and N477S (in *PRPF6*) were highly conserved in different species. A second change in *DNAJC5*, Leu115Arg, was also highly conserved.

Both changes in the genes *PRPF6* and *DNAJC5* occurred within ∼75 kb on chromosome 20 (Fig. 3). We conclude that they are physically linked on a rare haplotype. To further decide which gene represented the cause for ADKD we Sanger-sequenced all coding exons of the two genes in eight additional families (Table 1). We identified a Leu115Arg change in *DNAJC5* in one additional unrelated ADKD sample (see Table 1). This latter change occurred very close to the in-frame deletion Leu116del, in a highly conserved residue and is predicted to be pathological. This missense mutation was not present in 2,100 control subjects. No changes in *PRPF6* were identified in individuals unrelated to the Parry family.

Haplotype reconstruction with genetic markers in DNAJC5 showed that the studied affected individuals from the Parry family do not share haplotype with any of the families reported by Noskova et al [4] supporting the existence of a mutation hotspot rather than founder effect for the DNAJC5 deletion (Table 3). Haplotype analysis of individual BD319 also did not show overlap with haplotypes of individuals with the Leu115Arg changes reported by Noskova et al (Table 4).

**Table 3:** Haplotype reconstruction with genetic markers in *DNAJC5*.

	Leu116del					
	Parry VI:2	Parry V:27	Parry V:28	Parry associated haplotype	UCL519	P1
rs2065993	A/A	A/G	A/G	A	G/A	G/G
rs2247381	G/G	A/G	A/G	G	A/G	A/A
rs2427546	C/C	T/C	T/C	C	T/C	T/T
rs2427547	G/G	A/G	G/G	G	A/G	A/A
rs2427550	A/A	C/A	C/A	A	C/A	C/C
rs2427551	G/G	A/G	A/G	G	A/G	A/A
rs2427552	G/A	G/G	G/G	G	G/G	G/G
rs6011225	C/C	A/C	C/C	C	C/C	A/A
rs2427549	C/C	C/T	C/T	C	C/C	C/C
rs2427548	T/T	C/T	C/T	T	C/C	C/C
rs2427553	A/A	C/A	C/A	A	C/A	C/C
rs6062586	T/T	C/T	C/T	T	C/C	C/C
rs6122041	C/C	T/C	T/C	C	T/C	T/T
rs2427554	A/A	G/A	G/A	A	G/A	G/G
rs6011226	A/A	T/A	T/A	A	T/A	T/T
DNAJC6	Leu116del	Leu116del	Leu116del	Leu116del	Leu116del	Leu116del

Two informative homozygous discordant genotypes close to the Leu116del change indicate that the Parry family does not share a haplotype with either of the two families reported in Noskova et al supporting a mutational hotspot rather than a founder effect. Grey: Noskova et al [4] haplotypes; Red: Discordant homozygous genotypes; all markers appear in their genomic order from centromere to telomere.

**Table 4:** Haplotype reconstruction with genetic markers in *DNAJC5*.

Leu115Arg				
	BD319	UCL328	N1	UCL563
rs2065993	A/A	genotypes; all markers appear in their genomic order from centromere to telomere.A/A	G/G	G/G
rs2247381	G/G	G/G	A/A	A/A
rs2427546	C/C	C/C	T/T	T/T
rs2427547	G/G	G/G	A/G	A/A
rs2427550	A/A	A/A	C/C	C/C
rs2427551	G/G	G/G	A/A	A/A
rs2427552	G/A	G/A	G/G	G/G
rs6011225	C/C	n/a	A/C	C/C
rs2427549	C/C	n/a	C/C	C/C
rs2427548	T/T	n/a	C/C	C/C
rs2427553	A/A	C/C	C/C	C/C
rs6062586	T/T	C/C	C/C	C/C
rs6122041	C/C	C/C	T/T	T/T
rs2427554	A/A	A/A	G/G	G/G
rs6011226	A/A	A/A	T/T	T/T
DNAJC5	Leu115Arg	Leu115Arg	Leu115Arg	Leu115Arg

Three homozygous discordant genotypes close to the Leu115Arg change indicate that family BD319 does not share a haplotype with the three families reported in Noskova et al supporting a mutational hotspot rather than a founder effect. Grey: Noskova et al haplotypes; Red: Discordant homozygous genotypes; all markers appear in their genomic order from centromere to telomere.

## Discussion

We have performed exome sequencing on a large pedigree with ADKD and identified two highly significant changes in two neighboring genes on chromosome 20, which segregated with the phenotype. By screening additional ADKD patients we found an additional significant change in one family in the gene *DNAJC5*, but none in the gene *PRPF6*. We thus conclude, in agreement with the recent study by Noskova et al [4] that *DNAJC5* is a new gene causing ADKD in a subset of families with the disease. *DNAJC5* (MIM611203) codes for the protein Cystein String Protein alpna (CSPα). CSPα is highly conserved and is expressed in the brain. It associates with proteins SGTA and HSPA8 in a complex located on the synaptic vesicle surface that is believed to have a role in maintenance of the synapse function [8]. CSPα-deficient mice appear normal at birth, but at 2–4 weeks develop a fatal progressive disorder that includes muscle weakness and sensorimotor deficiency [9].

The additional identification of a significant change in *PRPF6* in the Parry family is of interest. PRPF6 is a pre-mRNA splicing factor important in the formation of the spliceosome. The encoded protein has been shown to bind to the androgen receptor thus potentially linking transcriptional activation and splicing. Mutations in *PRPF6* and other pre-mRNA splicing factors have been reported to cause rare forms of autosomal-dominant retinitis pigmentosa [7]. Retinitis pigmentosa is not a consistent feature of ADKD, but several family members of the Parry family have been reported with apparently functional vision problems (see methods and reference 5). Given that both changes in *DNAJC5* and *PRPF6* were not identified in a large number of controls and their complete co-segregation within the extended pedigree, it can be assumed that they fall on the same rare haplotype. Since limited information regarding the visual status of members of the Parry family exist, it was impossible to follow visual phenotype segregation in the family. Therefore further clinical studies of the family are needed in order to reach a definite conclusion about the role of the PRPF6 sequence change as a phenotype modifier.

We report a detailed clinical description of the Pary family proband in order to provide a detailed *DNAJC5* deficiency-associated phenotype. Several conclusions can be made from the reported phenotypes of the proband and her relatives.. Firstly, no reliable confirmation of Kufs disease in individuals with *DNAJC5* mutations could be made by studying peripheral cells for lysosomal inclusions in the earlier stages of the disorder. It seems that the EM studies of peripheral cells in *DNAJC5* mutation-negative individuals tend to be more informative, compared to those in mutation-positive individuals, but this remains to be confirmed in further studied (see Table 2). This emphasizes the usefulness of conducting a molecular study for *DNAJC5* mutations when ADKD is suspected. Such testing will be likely available soon. Secondly, psychiatric manifestations may be the first presentation in *DNAJC5* positive individuals. The proband of the Parry family presented with such manifestations long before other symptoms were noted. While the current study was underway, mutations in *DNAJC5* were reported in 5 ADKD individuals [4]. 20 individuals from different families were included in this study. However only seven had a clear autosomal dominant inheritance. Also, results of confirmatory neuropathological studies of brain tissue were not reported for the individuals negative for *DNAJC5* mutations. It is thus difficult to determine the exact proportion of ADKD individuals with *DNAJC5* mutations from the report of Noskova et al. since no reported laboratory confirmation of ADKD in most mutation-negative individuals/families was available.

Our study included only individuals/families with known autosomal dominant inheritance or sporadic individuals thus minimizing the likelihood of families with recessive inheritance. Most importantly, all affected individuals in the present study had confirmation of the diagnosis of Kufs disease by neuropathological studies of the brain. In our set two out of nine individuals/families (22%) had *DNAJC5* mutations. If we only consider strictly known autosomal dominant families then 1 out of 5 families (20%) had a *DNAJC5* mutation. It is therefore clear that other genes are involved in the majority of ADKD cases (up to 80% in our sample).

In the group of NCL, ADKD is classified as NCL 4B (MIM 162350). We propose that the type of ADKD that is associated with mutations in *DNAJC5* is classified as NCL 4B1.

In summary, we have identified variants in *DNAJC5* as cause for ADKD in approximately 20% of autosomal-dominant families, confirming a recent report by Noskova et al [4]. We conclude that additional genetic studies will be necessary to identify the remaining genes for ADKD and fully characterize the molecular genetic spectrum of this disease.

### Limitations

It is not possible to rule out with certainty recessive inheritance in our sporadic cases, but if only individuals with clearly documented autosomal-dominant inheritance in our study are considered, the prevalence of *DNAJC5* mutation-positive individuals does not change significantly compared to the value using all individuals studied (about 20%). It thus appears that most genetic mutations associated with ADKD remain to be identified.
