# Correction: Mutations in the Gene *DNAJC5* Cause Autosomal Dominant Kufs Disease in a Proportion of Cases: Study of the Parry Family and 8 Other Families

**DOI:** 10.1371/annotation/26d7eb64-ccd2-41db-b1aa-7cdc8c1eff95

**Published:** 2012-09-05

**Authors:** Milen Velinov, Natalia Dolzhanskaya, Michael Gonzalez, Eric Powell, Ioanna Konidari, William Hulme, John F. Staropoli, Winnie Xin, Guang Y. Wen, Rosemary Barone, Scott H. Coppel, Katherine Sims, W. Ted Brown, Stephan Züchner

The authors would like to make a correction regarding one of the individuals reported in the study. Suspected specimen mislabeling for individual KUF04SR (see Table 2), resulted in reporting of a false-negative result for DNAJC5 mutation in this individual. This subject was independently shown to harbor a heterozygous c.346_348delCTC/p.Leu116del mutation in DNAJC5. 

